# The Usefulness of Quantitative Analysis of Blood-Brain Barrier Disruption Measured Using Contrast-Enhanced Magnetic Resonance Imaging to Predict Neurological Prognosis in Out-of-Hospital Cardiac Arrest Survivors: A Preliminary Study

**DOI:** 10.3390/jcm9093013

**Published:** 2020-09-18

**Authors:** Ho Il Kim, In Ho Lee, Jung Soo Park, Da Mi Kim, Yeonho You, Jin Hong Min, Yong Chul Cho, Won Joon Jeong, Hong Joon Ahn, Changshin Kang, Byung Kook Lee

**Affiliations:** 1 Department of Emergency Medicine, Chungnam National University Hospital, 282, Munhwa-ro, Jung-gu, Daejeon 35015, Korea; laverhi@naver.com (H.I.K.); yyh1003@hanmail.net (Y.Y.); boxter73@naver.com (Y.C.C.); gardenjun@hanmail.net (W.J.J.); jooniahn@hanmail.net (H.J.A.); changsiny@naver.com (C.K.); 2 Department of Radiology, College of Medicine, Chungnam National University, 266, Munhwa-ro, Jung-gu, Daejeon 35015, Korea; leeinho1974@hanmail.net (I.H.L.); damirad@cnuh.co.kr (D.M.K.); 3 Department of Emergency Medicine, College of medicine, Chungnam National University, Daejeon 35015, Korea; shiphid@hanmail.net; 4 Department of Emergency Medicine, Chonnam National University Medical School, Gwangju 61469, Korea; bbukkuk@hanmail.net

**Keywords:** heart arrest, prognosis, magnetic resonance imaging

## Abstract

We aimed to evaluate neurological outcomes associated with blood-brain barrier (BBB) disruption using contrast-enhanced magnetic resonance imaging (CE-MRI) in out-of-hospital cardiac arrest (OHCA) survivors. This retrospective observational study involved OHCA survivors who had undergone CE-MRI for prognostication. Qualitative and quantitative analyses were performed using the presence of BBB disruption (pBD) and the BBB disruption score (sBD) in CE-MRI scans, respectively. For the sBD, 1 point was assigned for each area of BBB disruption, and 6 points were assigned when an absence of intracranial blood flow due to severe brain oedema was confirmed. The primary outcome was poor neurological outcome at 3 months (defined as cerebral performance categories 3–5). We analysed 46 CE-MRI brain scans (27 patients). Of these, 15 (55.6%) patients had poor neurological outcomes. Poor neurological outcome group patients showed a significantly higher proportion of pBD than those in the good neurological outcome group (22 (88%) vs. 6 (28.6%) patients, respectively, *p* < 0.001) and a higher sBD (5.0 (4.0–5.0) vs. 0.0 (0.0–1.0) patients, *p* < 0.001). Poor neurological outcome predictions showed that the sBD had a significantly better prognostic performance (area under the curve (AUC) 0.95, 95% confidence interval (CI) 0.84–0.99) than the pBD (AUC 0.80, 95% CI 0.65–0.90). The sBD cut-off value was >1 point (sensitivity, 96.0%; specificity, 81.0%). The sBD is a highly predictive and sensitive marker of 3-month poor neurological outcome in OHCA survivors. Multicentre prospective studies are required to determine the generalisability of these results.

## 1. Introduction

Cardiac arrest (CA) is a common cause of death and disability [1]. Among more than 300,000 CA events that occur each year in the United States, the rate of survival to hospital discharge from out-of-hospital cardiac arrest (OHCA) has been reported to be <10% [2]. Even when return of spontaneous circulation (ROSC) is achieved, approximately 30% of survivors have permanent brain damage [3]. Several prognostic methods have been suggested for predicting neurologic outcome. All prognostic tools are recommended 48–72 h after CA except for brain computed tomography (CT) and status myoclonus (within 24 h) [4]. However, sedatives and neuromuscular blocking agent might result in misleading outcome predictions regarding the presence of myoclonus. Current prognostication guidelines suggest performing brain magnetic resonance imaging (MRI) 2–5 days after ROSC [5]. However, recent evidence has shown that MRI is better than brain CT at predicting neurological outcome within 3 h after ROSC [6,7,8].

Brain injury following cardiopulmonary resuscitation (CPR) is a significant cause of morbidity in survivors [9]. One of the most serious complications following CA is brain oedema, which is associated with poor neurological outcome and death [3]. Following cerebral ischemia-reperfusion, pathological alterations to the blood-brain barrier (BBB) play a decisive role in the ensuing formation of oedema [10]. As a result, BBB disruption is a major factor leading to permanent cerebral oedema after CA and resuscitation [11]. However, the relationship between BBB disruption after CA and neurological prognosis is rarely discussed in clinical studies, in contrast to animal studies. Our previous study is the first clinical study wherein we report that severe BBB disruption onset timing and severe BBB disruption are strongly associated with poor neurological outcomes; however, we did not observe a relationship between neurological outcome and the degree of BBB disruption [9]. Therefore, determining the degree of BBB disruption is likely to be very helpful in predicting the prognosis of patients with CA. Many studies have been conducted on BBB disruption.

The cerebrospinal fluid (CSF)-serum albumin quotient (Q_A_), calculated using CSF/serum-albumin is a gold standard numerical indicator used to determine the functional assessment of BBB disruption [12]. Contrast-enhanced magnetic resonance imaging (CE-MRI) has also been confirmed as a useful imaging test to determine the degree of BBB disruption, and this indicator can be visually confirmed [13].

To date, no study has reported the use of CE-MRI to measure BBB disruption as a prognostic factor for patients with CA. In addition, the relationship between the Q_A_ and the degree of BBB disruption measured using CE-MRI in patients with CA has not been identified. Therefore, we aimed to evaluate the usefulness of a quantitative analysis of BBB disruption measured using CE-MRI to predict neurological prognosis in OHCA survivors.

## 2. Patients and Methods

### 2.1. Study Design and Patients

In this retrospective observational study, we used prospectively collected data derived from adult comatose OHCA survivors treated with target temperature management (TTM) at Chungnam National University Hospital in Daejeon, Korea, between April 2019 and February 2020. The Institutional Review Board of Chungnam National University Hospital approved this study (CNUH-2020-06-022).

Our inclusion criteria comprised adult OHCA survivors (age, ≥18 years) who were unconscious (Glasgow Coma Scale score, ≤8) after ROSC and who had been treated with TTM. Exclusion criteria comprised patients: (1) aged <18 years; (2) with traumatic CA; (3) with an interrupted TTM (because of transfer from another facility or due to hemodynamic instability (<60 mmHg mean arterial pressure or <90 mmHg systolic blood pressure despite ≥6 h of the vasopressor support); (4) not eligible for TTM (i.e., because of intracranial haemorrhage, active bleeding, a known terminal illness, or a poor pre-CA neurological status), and; (5) who had been administered extracorporeal membrane oxygenation (ECMO).

### 2.2. Target Temperature Management Protocol

The patients had been managed according to our previously published TTM protocol [14]. A target temperature was maintained at 33 °C for 24 h using feedback-controlled surface-cooling devices (Artic Sun^®^ Energy Transfer Pads™; Medivance Corp, Louisville, CO, USA). Midazolam (0.05 mg/kg intravenous bolus, followed by a titrated intravenous continuous infusion of 0.05–0.2 mg/kg/h) and cisatracurium (0.15 mg/kg intravenous bolus, followed by an infusion of up to 0.3 mg/kg/h) were administered for sedation and to control shivering. Electroencephalography was performed if there was a persistent deterioration in a patient’s level of consciousness, involuntary movements, or seizure. If there was evidence of electrographic seizure or a clinical diagnosis of seizure, an anti-epileptic drug was administered, namely, levetiracetam (loading dose 2 g bolus intravenously and maintenance dose, 1 g bolus twice daily, intravenously). All patients were treated with standard intensive care according to our institutional intensive care unit protocol.

### 2.3. Data Collection and Primary Outcome

The following data were collected from the database: age, sex, presence of a witness at the time of collapse, bystander CPR, first monitored rhythm, aetiology of CA, time from collapse to CPR (no flow time), time from CPR to ROSC (low flow time), time from ROSC to first and second MRI scan, and neurological outcome after CA.

The primary endpoint of this study was neurological outcome 3 months after CA. We measured neurological outcome 3 months after ROSC using the Glasgow Pittsburgh cerebral performance category (CPC) scale, either via face-to-face interviews or structured telephone interviews. Phone interviews were conducted by an emergency physician who had been fully informed of the protocol and was blinded to patient prognoses, the CE-MRI findings, and the Q_A_. A poor neurological outcome was defined as a CPC score of 3, 4, or 5.

### 2.4. Q_A_ measurement

CSF was obtained via lumbar catheter drainage, and serum was collected through venepuncture. A lumbar catheter insertion was performed using a Hermetic^TM^ lumbar catheter accessory kit (Integra Neurosciences, Plainsboro, NJ, USA), with the patient lying in a lateral decubitus position with hips and knees flexed. CSF albumin and serum albumin samples were both obtained at the same time within 6 h after ROSC and between 72 h and 96 h after ROSC. Albumin analysis was performed using a TBA-2000FR (Canon Medical Systems Corporation, Otawara, Japan). The Q_A_ was calculated using the following formula: [albumin_CSF_]/[albumin_serum_]. The degree of BBB disruption was defined as follows: Q_A_ > 0.007 (more than mild), >0.01 (more than moderate), and ≥0.02 (severe).

### 2.5. Qualitative and Quantitative Analyses of BBB Disruption Using CE-MRI

In this study, we undertook qualitative and quantitative analyses using the presence of BBB disruption (pBD) and the BBB disruption score (sBD) in CE-MRI scans, respectively. Our institution has a standardised CE-MRI protocol for nontraumatic OHCA survivors. If a patient’s condition was haemodynamically stable and the patient’s family consented to an MRI scan, all OHCA survivors underwent two MRI scans. The first MRI was obtained within 6 h after ROSC, and the second was obtained between 72 h and 96 h after ROSC. CE-MRI brain images were obtained using a 3T scanner (Achieva 3T, Philips Medical System, Andover, Netherlands). The Q_A_ measurement and the CE-MRI brain images were both obtained simultaneously. The protocol for using the 3T scanner included a precontrast fluid attenuated inversion recovery (FLAIR) (TR/TE, 11,000/125 ms; section thickness, 5 mm; section gap, 1.5 mm; FOV, 220 × 220 mm; matrix, 316 × 184, number of slices, 24, and; number of excitations, 2) and a post-contrast FLAIR (TR/TE, 11,000/110 ms; section thickness, 5 mm; section gap, 1.5 mm; FOV, 220 × 220 mm; matrix, 316 × 184, number of slices, 24, and; number of excitations, 2) after the administration of gadobutrol (Gadovist, Bayer Healthcare, Berlin, Germany) at a dose of 0.1 mmol/kg (or up to a total dose of 10 mL) and an injection rate of 0.5–1 mL/sec. The affected regions of the brain were selected by comparing each image using a slice-wise method.

**Figure 1:**
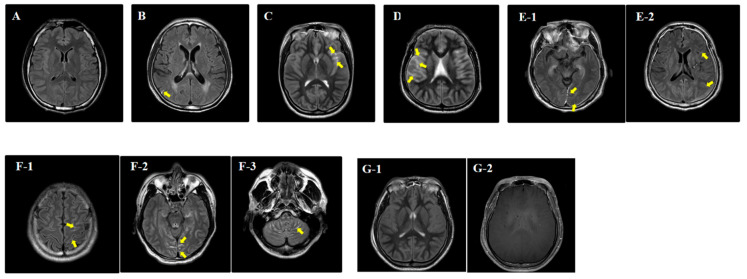
The blood-brain barrier disruption score using a post-contrast fluid-attenuated inversion recovery image. (**A**) Leptomeningeal enhancement in the subarachnoid space is not visible; a score of 0. (**B**) Leptomeningeal enhancement in the right parietal sulcus is visible; a score of 1. (**C**) Leptomeningeal enhancement in the left sylvian fissure (lateral sulcus separating the frontal lobe from the temporal lobe) is visible; a score of 2. (**D**) Leptomeningeal enhancement in the right frontal, parietal and temporal sulci is visible; a score of 3. (**E-1**) Leptomeningeal enhancement in the left temporo-occipital sulci and, (**E-2**) left fronto-parietal sulci is visible; a score of 4. (**F-1**) Leptomeningeal enhancement in the left fronto-parietal sulci, (**F-2**) left temporo-occipital sulci and, (**F-3**) cerebellar folia is visible; a score of 5. (**G-1**) Leptomeningeal enhancement is not visible in the subarachnoid space and, (**G-2**) the intracranial blood flow is absent in the contrast enhanced T1 weighted MR; a score of 6.

The obtained CE-MRI brain images were interpreted by two board-certified neuroradiologists who were blinded to patient information. Gadobutrol was used as a contrast agent during the CE-MRI examination. The pBD on CE-MRI was identified through confirming gadobutrol leakage on postcontrast FLAIR scans compared to precontrast FLAIR scans. The pBD was evaluated according to brain regions (frontal, parietal, temporal, occipital, and cerebellum). One point was assigned for each area where BBB disruption was present, and 6 points were assigned when the absence of intracranial blood flow due to severe brain oedema was confirmed. Depending on the scoring system, a possible score could range from 0 to 6 points (Figure 1).

### 2.6. Using CE-MRI to Analyse the Relationship between Q_A_ and BBB Disruption

We undertook a receiver operating characteristic (ROC) curve analysis to determine the performance of the Q_A_ in predicting BBB disruption using CE-MRI. BBB disruption was assessed using the sBD, which was redefined as binary. If the sBD was equal to or exceeded the cut-off value of the highest area under the ROC (AUROC) capacity predicting poor neurological outcome at 3 months, this was classified as BBB disruption.

### 2.7. Statistical Analysis

Categorical variables are presented as frequencies and percentages, and continuous variables are presented as means and standard deviations, or as median and interquartile ranges, depending on the normality of the distribution. The normal distribution of data was analysed using a Shapiro–Wilk test. We compared categorical variables between the groups using Pearson’s chi-squared- or Fisher’s exact tests, as appropriate. We compared continuous variables between the groups using independent t- or Mann–Whitney U tests, as appropriate. AUROC analysis was performed to examine the relationship between the Q_A_ and BBB disruption using CE-MRI and the prognostic performance of the Q_A_, pBD, and sBD for poor neurological outcome at 3 months after ROSC. The optimum cut-off values were determined using Youden’s index (sensitivity + specificity – 1). Subsequently, we used a Delong test to determine differences in the relationship and prognostic performance. Data were analysed using SPSS for Windows, version 21 (IBM Corp., NY, NY, USA). ROC curves were calculated using MedCalc version 15.2.2 (MedCalc Software, Mariakerke, Belgium). A two-sided significance level of 0.05 was used to indicate statistical significance.

## 3. Results

### 3.1. Patient Demographics

During the study period, of 39 patients with OHCA who had been admitted to the intensive care unit for TTM, 1 had not undergone a first or a second MRI, 1 had undergone an MRI within >6 h (first scan), 1 had undergone ECMO treatment, and a different scanning protocol (such as a diffusion-weighted image (DWI)-MRI only) had been used for 9 patients. As a result, 27 patients were enrolled in the study, of whom 19 (70.4%) had undergone two CE-MRI scans.

Table 1 shows the baseline and CA characteristics according to neurological outcome. At 3 months after ROSC, 15 (55.6%) patients had a poor neurological outcome. The median age was 60.0 (40.0–70.0) years, and 21 patients (77.8%) were men. The first brain MRI scans were obtained at a median of 2.6 (1.9–3.9) h after ROSC and the second brain MRI scans were acquired at a median of 76.7 (75.2–76.7) h after ROSC. Patients with poor neurological outcomes were less likely to have had a witnessed CA, bystander CPR, and shockable rhythms. Moreover, they had longer low flow and no flow times. Table 2 shows the individual values for Q_A_, pBD and sBD for a total 27 patients.

### 3.2. A Comparison of pBD and sBD Using CE-MRI to Predict Neurological Outcome

**Figure 2:**
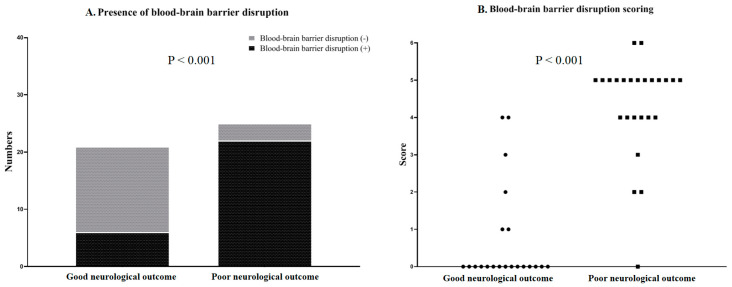
Comparisons between the proportion and the score of blood-brain barrier disruption between the good and the poor neurological outcome groups. (**A**) In the poor neurological outcome group, the proportions of BBB disruption were significantly higher than those in the good neurological outcome group (*p* < 0.001). (**B**) In the poor neurological outcome group, the scores of BBB disruption were significantly higher than those in the good neurological outcome group (*p* < 0.001).

**Figure 3:**
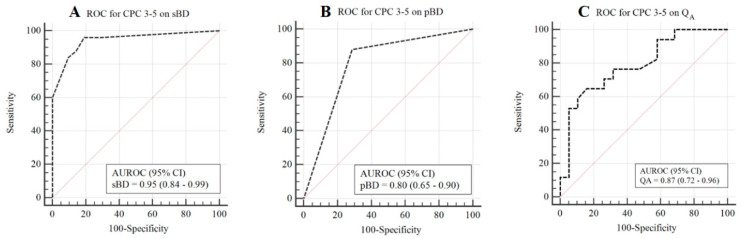
Association of sBD, pBD and Q_A_ with poor neurological outcomes. (**A**) Receiver operating characteristic curve for poor neurological outcome on sBD is shown on the panel. (**B**) Receiver operating characteristic curve for poor neurological outcome on pBD is shown on the panel. (**C**) Receiver operating characteristic curve for poor neurological outcome on Q_A_ is shown on the panel.

A total of 46 CE-MRI scans were acquired in 27 patients. Of these CE-MRI scans, 21 (45.7%) showed a good neurological outcome and 25 (55.3%) showed a poor neurological outcome. The locations of BBB disruption and each score for the 46 postcontrast FLAIR scans according to the BBB disruption scoring system are summarised in Table 3. In total, 16 scans had scores of 0, and 13 scans had scores of 5. The poor neurological outcome group showed a significantly higher proportion of pBD and higher sBD scores than the good neurological outcome group (22 (88%) vs. 6 (28.6%) patients, *p* < 0.001 and 5.0 (4.0–5.0) vs. 0.0 (0.0–1.0), *p* < 0.001; respectively; Figure 2). A significantly higher AUROC capacity to predict poor neurological outcome at 3 months was found for sBD than for pBD (0.95 (95% CI 0.84–0.99) vs. 0.80 (95% CI 0.65–0.90), *p* = 0.015), respectively (Figure 3). The cut-off value of the sBD was >1 point, with sensitivity and specificity of 96.0% and 81.0%, respectively (Table 4).

### 3.3. A Comparison of the Relationship between the Q_A_ and sBD and their Ability to Predict Neurological Outcome

Table 4 shows the relationship between the Q_A_ and neurological outcome. In total, 36 Q_A_ were acquired in 27 patients. The Q_A_ was 0.02 (0.009–0.0546) in the poor neurological outcome group and 0.0083 (0.0053–0.0131) in the good neurological outcome group (*p* = 0.003). According to the level of BBB disruption between the good and poor neurological outcome groups, there were 11 (57.9%) vs. 15 (88.2%) patients with more than mild BBB disruption (*p* = 0.065), 5 (26.3%) vs. 12 (70.6%) patients with more than moderate BBB disruption (*p* = 0.018), and 1 (5.3%) vs. 8 (47.1%) patients with severe BBB disruption, respectively, (*p* = 0.006) (Table 5). In addition, the ability to predict poor neurological outcomes in the sBD was significantly higher than in the Q_A_ (*p* = 0.013) (Table 4).

## 4. Discussion

In this retrospective observational study, qualitative (pBD) and quantitative (sBD) analyses of BBB disruption using CE-MRI showed a significant difference between the good and poor neurological outcome groups after 3 months in OHCA survivors treated with TTM. Quantitative analysis predicted prognostic performance better than qualitative analysis. In addition, the sBD showed a significantly higher neurological outcome predictive performance than the Q_A_.

Transient global ischemic brain injury from CA may result in increased permeability and disruption of BBB tight junctions. In particular, leukocytes activated during reperfusion interact with endothelial cells and plug capillaries and interfere with the BBB through the release of neutrophil-derived oxidants and proteolytic enzymes [15,16]. Several previous studies involving patients with ischaemic stroke have reported that BBB disruption during cerebral reperfusion may lead to the development of vasogenic oedema and a poor neurological outcome [17,18,19,20,21]. In addition, our previous study also reported that severe BBB disruption, indicated as a Q_A_ ≥ 0.02, occurred within the first 24 h after ROSC in a patient group with poor neurological outcomes who had been treated with TTM [9]. However, the Q_A_ has limitations in routine clinical use because CSF is required for the Q_A._

Under normal physiological conditions, contrast-enhanced FLAIR images cannot detect BBB disruption during reperfusion, because contrast agents do not cross the intact BBB [16]. In a study using postcontrast FLAIR images (termed hyperintense acute perfusion marker, HARM) to characterise early BBB disruption, Steven and Lawrence reported that HARM was found in 47 of 144 patients with ischaemic stroke, and was related to haemorrhagic transformation and poor clinical outcome [17]. In our study, the poor outcome group showed more BBB disruption than the good outcome group (88% vs. 28.6%, respectively). In addition, our study showed that the sBD was superior to the pBD in predicting neurological outcome. Two explanations are possible for these results. First, an absence of intracranial blood flow due to severe brain oedema was observed in 2 patients, who both showed a poor neurological outcome. These patients had the highest score, i.e., 6 in the sBD, but had negative results in terms of the pBD. Second, a recent retrospective study of 128 patients that investigated the relationship between BBB disruption and neurological complications in patients undergoing endovascular treatment of unruptured intracranial aneurysms reported that an increasing extent of BBB disruption was associated with the development of a neurological event [22]. In this study, BBB disruptions were noted in 2 patients who had an sBP score of 1 point, both of whom had a good neurological outcome. When compared to the cut-off value (sBD > 1, pBD; presence), patients are expected to have a good neurological outcome with an sBD, but a poor neurological outcome with a positive pBD.

The Q_A_ has been widely accepted as the gold standard for the functional assessment of BBB disruption [12]. However, in this study, CE-MRI was used to determine the presence and degree of BBB disruption. While CE-MRI is a more costly procedure than obtaining a Q_A_, it has the advantage of excellent visualisation and clinical application for assessing BBB disruption and hypoxic ischaemic-reperfusion brain injury [18]. In addition, in this study, the sBD showed better predictability for poor neurological outcome at 3 months after ROSC than the Q_A_. These results may be explained as follows. First, gadobutrol has a molecular weight of 550 Dalton (Da) [22], which is a smaller molecular weight than that of albumin (molecular weight, 66,700 Da) [23]. If BBB disruption occurs, both substances pass through the BBB; however, gadobutrol, which is approximately 100 times smaller in size, is likely to be more permeable and sensitive than albumin. Second, since haemolysis may increase albumin levels, it is also possible that some patients have increased CSF albumin levels without necessarily reflecting brain injury due to haemolysis during the lumbar catheter insertion.

In addition, Son et al.’s recent study showing poor neurological outcome using DWI-MRI conducted before TTM after ROSC reported a high signal intensity in 22 of 33 (66.7%) patients [8]. One previous study reported that, while DWI is not noticeable in most patients with transient ischemic attack, BBB interruptions may exist. Therefore, HARM can be an additional useful diagnostic tool for assessing patients with acute ischemic stroke [14]. In future, it may be necessary to examine whether combining the sBD and DWI-MRI brain scans provides better predictions concerning neurological outcomes in CA survivors.

This study had several limitations. First, since this is a single-centre retrospective study with a small number of patients, it may affect the selected threshold or other statistical outcome of this work; therefore, a multicentre prospective study is required to enhance the generalisability of the findings. Second, patients were evaluated according to brain CE-MRI and/or CSF and serum albumin levels. However, CE-MRI scans before TTM and lumbar catheter insertion are rare in clinical practice and are very complex to apply; hence, they are generally not used. Third, bias was possible due to the treating physicians being aware of the CSF results or the serum albumin levels. Fourth, CE-MRI scans were performed in all patients, but the inability to obtain CSF albumin levels in some patients may have contributed to bias. In addition, the inability to obtain CSF albumin levels in patients with suspected brain death may have a significant effect on the identification of the prognostic performance of the Q_A_. While there was a small number of patients for whom we could not obtain a Q_A_, given the ratio in terms of the total number of patients, this could have had a considerable effect on our results. Fifth, we used a manual scoring system in this study. However, in future studies involving a larger population, it would be appropriate to use “post-pre subtraction image processing tool” to make more objective and quicker judgments.

## 5. Conclusions

Using CE-MRI, the sBD was shown to be a significantly predictive marker of poor neurological outcome at 3 months in OHCA survivors compared to the pBD and the Q_A_. Multicentre prospective studies are required to determine the generalisability of these results.

## Figures and Tables

**Table 1:** Baseline demographics and clinical characteristics of 27 patients.

Characteristics	Cohort (*n* = 27)	Good Outcome (*n* = 12)	Poor Outcome (*n* = 15)	*p*-Value
Age, years, median (IQR)	60.0 (40.0–70.0)	60.5 (46.5–69.5)	60 (40.0–74.0)	0.764
Sex, male, *n* (%)	21 (77.8)	11 (91.7)	10 (66.7)	0.182
Arrest characteristics				
Witness arrest, *n* (%)	18 (66.7)	11 (91.7)	7 (46.7)	0.014
Bystander CPR, *n* (%)	17 (63.0)	11 (91.7)	6 (40.0)	0.006
Shockable rhythm, *n* (%)	4 (14.8)	4 (33.3)	0 (0.0)	0.015
Cardiac aetiology, *n* (%)	11 (40.7)	7 (58.3)	4 (26.7)	0.096
No flow time, min (IQR)	3.5 (0.0–25.5)	0 (0.0–2.5)	21 (7.8–36.3)	0.005
Low flow time, min (IQR)	23.0 (9.0–31.0)	15 (6.5–23.5)	30 (20.0–39.0)	0.017
ROSC to first MRI time, hr (IQR)	2.62 (1.87–3.86), 22 *	2.15 (1.63–3.44), 10 *	2.75 (2.21–5.41), 12 *	0.129
ROSC to second MRI time, hr (IQR)	76.72 (75.19–76.72), 24 *	75.67 (74.47–77.87), 11 *	77.18 (75.97–81.37), 13 *	0.111

IQR, interquartile range; CPR, cardiopulmonary resusciation; ROSC, return of spontaneous circulation; MRI, magnetic resonance imaging; * Number of MRI scan in the analysis.

**Table 2:** Individual values for Q_A_, pBD and sBD for a total of 27 patients.

Case Number	Q_A_		pBD		sBD	
	First	Second	First	Second	First	Second
Patient 1	0.0132	0.0161	Absence	Presence	0	4
Patient 2	0.0094		Absence	Absence	0	0
Patient 3	0.0083	0.0091	Presence	Presence	1	1
Patient 4		0.0065		Presence		4
Patient 5			Presence	Absence	5	6
Patient 6		0.0176	Absence	Presence	0	4
Patient 7	0.0054		Absence		0	
Patient 8	0.0083	0.0593	Presence	Presence	5	5
Patient 9	0.0345		Presence	Presence	4	4
Patient 10	0.0097		Presence	Absence	4	6
Patient 11	0.0500	0.1188	Presence	Presence	2	5
Patient 12	0.0100	0.0133	Absence	Absence	0	0
Patient 13			Presence	Presence	4	3
Patient 14	0.0242	0.1000	Presence	Presence	5	5
Patient 15	0.0091	0.0057	Absence	Absence	0	0
Patient 16	0.0050	0.0061	Absence	Absence	0	0
Patient 17		0.0049		Absence		0
Patient 18		0.0071		Presence		4
Patient 19	0.0161	0.0615	Presence	Presence	5	5
Patient 20	0.0083	0.0029	Presence	Absence	3	0
Patient 21	0.0103		Presence		5	
Patient 22			Absence	Presence	0	2
Patient 23	0.0056	0.0200	Presence	Presence	5	5
Patient 24	0.0167		Presence		5	
Patient 25		0.0621		Presence		2
Patient 26		0.0250		Presence		5
Patient 27	0.0053	0.0029	Absence	Absence	0	0

First: MRI or Q_A_ was obtained within 6 h after ROSC; Second: MRI or Q_A_ was obtained between 72 h and 96 h after ROSC. Q_A_, cerebrospinal fluid-serum albumin quotient; pBD, presence of blood-brain barrier disruption; sBD, blood-brain barrier disruption score

**Table 3:** Location and numbers of BBB disruption, scored according to BBB disruption scoring system in 46 CE-MRI scans.

Score	Number	Location of BBB Disruption
0	16	None
1	2	Parietal lobe, *n* = 2
2	3	Frontal lobe + Parietal lobe, *n* = 1 Temporal lobe + Occipital lobe, *n* = 2
3	2	Frontal lobe + Parietal lobe + Temporal lobe, *n* = 1 Parietal lobe + Temporal lobe + Occipital lobe, *n* = 1
4	8	Frontal lobe + Parietal lobe + Temporal lobe + Occipital lobe, *n* = 8
5	13	Frontal lobe + Parietal lobe + Temporal lobe + Occipital lobe + Cerebellum, *n* = 13
6	2	No internal carotid artery flow, *n* = 2

BBB, blood-brain barrier; CE-MRI, contrast-enhanced magnetic resonance imaging.

**Table 4:** Prognostic performances of sBD, pBD and Q_A_ for neurological outcome.

Characteristics	AUR (95% CI)	Cut-off	Sensitivity	Specificity	PPV	NPV	*p*-Value for AUC Comparison
sBD	0.95 (0.84–0.99)	>1	96.0	81.0	80.0	93.7	Reference
pBD	0.80 (0.65–0.90)	Presence	88.0	71.4	78.6	83.3	0.015
Q_A_	0.87 (0.72–0.96)	>0.0133	66,7	100.0	100.0	68.2	0.013

AUR, the area under the receiver operating characteristic curve; PPV, positive predictive value; NPV, negative predictive value; sBD, blood-brain barrier disruption score; pBD, presence of blood-brain barrier disruption; Q_A_, cerebrospinal fluid-serum albumin quotient.

**Table 5:** Relationship between cerebrospinal fluid/serum albumin quotient and neurological outcome measured in 36 samples.

Characteristics	Good Neurological Outcome (*n* = 19)	Poor Neurological Outcome (*n* = 17)	*p*-value
Total cohort, median (IQR)	0.0083 (0.0053–0.0131)	0.02 (0.009–0.0546)	0.003
More than mild BBB disruption, *n* (%)	11 (57.9%)	15 (88.2%)	0.065
More than moderate disruption, *n* (%)	5 (26.3%)	12 (70.6%)	0.018
Severe BBB disruption, *n* (%)	1 (5.3%)	8 (47.1%)	0.006

IQR, interquartile range.

## Abbreviations

BBB	blood-brain barrier
CE-MRI	contrast-enhanced magnetic resonance imaging
OHCA	out-of-hospital cardiac arrest
pBD	presence of blood-brain barrier disruption
sBD	blood-brain barrier disruption score
AUC	area under the curve
CI	confidence interval
CA	cardiac arrest
ROSC	return of spontaneous circulation
CT	computed tomography
CPR	cardiopulmonary resuscitation
CSF	cerebrospinal fluid
Q_A_	albumin quotient
TTM	target temperature management
ECMO	extracorporeal membrane oxygenation
CPC	cerebral performance category
FLAIR	fluid attenuated inversion recovery
ROC	receiver operating characteristic
AUROC	area under the ROC
MRI	magnetic resonance imaging
DWI	diffusion-weighted image
HARM	hyperintense acute perfusion marker
